# Unqualified Dispensers

**Published:** 1899-11-25

**Authors:** 


### The Hospital

A Journal of

The Medical Sciences and Hospital Administration.

Vol. XXVII.—No. 687. Edited by Sir Henry Burdett, K.C.B., and Solomon C. Smith, M.D., M.R.C.P. [November 25, 1899.

### UNQUALIFIED DISPENSERS.

The General Medical Council, at its meeting next week, will be called upon to consider a question of much practical importance to a large number of country practitioners who supply medicines to their patients. It is well known that the Council, after having for some years given warning of its intentions, in such a manner as to afford every facility for both principals and assistants to accommodate themselves to the change, has lately expressed its determination to regard the employment of unqualified assistants, as substitutes for their principals in matters requiring professional knowledge and skill, as a deliberate fraud upon the patient, and, therefore, as amounting to "infamous conduct in a professional respect"; and it is difficult to see how this conclusion can be avoided. No one is more justly offended by the independent practice of an unqualified person than the qualified practitioner in his vicinity, and it is manifestly inconsistent for the latter to assume the privilege of employing an equally unqualified person himself, and of permitting this person to deal unaided with serious cases of illness or accident. The Council has been vainly urged to attempt the obvious impossibility of defining the limits within which, and the purposes for which, the employment of an unqualified assistant may be legitimate; but it is abundantly manifest that there would seldom be any difficulty in determining, in any given case, whether the line of propriety had been overpassed. A definition would only serve to afford facilities for evasion, and the Council can only undertake to consider on its merits any charge of the improper employment of an unqualified assistant which may come before them. The result of the action taken by the Council has already been exceedingly beneficial, although, like most changes, it has in some instances appeared to be productive of hardship. A certain proportion, probably not a large proportion, of unqualified assistants were men who had been prevented from completing their education by adverse circumstances beyond their control; and who, having derived from their interrupted studies and their partial knowledge a means of earning a living, have displayed, to the satisfaction of their employers, a fair capacity for turning what they had learnt to good use at the bedside, and for learning more as opportunities were afforded them. It is probably not unfair to say that the great majority were more or less wastrels. The practical disappearance of this class has opened fresh opportunities of occupation and of experience to large numbers of recently qualified men, and has definitely raised the standard of remuneration for young practitioners, while at the same time it has discouraged the attempts, once too common, to conduct by the aid of assistants a practice really too large or too widely extended to be within the reasonable capacity of any principal. An endeavour is now being made to induce the Council to take action for the suppression of unqualified dispensers. A person named Oglesby, a dentist at Barnsley, and a Mr. Cooper, who is a druggist in London, wrote under the same date to the Privy Council, to call attention to an alleged death at Heaton Norris, said to have been caused by the error of an incompetent dispenser in the employment of a local medical man; and their letters were referred by the Privy Council to the Medical Council, which appointed a committee to report at the coming session upon the question. The action of Mr. Cooper appears to have been prompted by a desire that those only should be employed as dispensers who have been declared by the Pharmaceutical Society to be qualified, he perhaps being ignorant that the Society of Apothecaries, under the Act of 1815, is entitled to grant a certificate of competence as a dispenser, and that this certificate is held by many persons of both sexes who have undergone the required curriculum of study and passed the prescribed examination. There can be no doubt that it would be good policy on the part of any medical man who supplies medicines, and whose practice is sufficiently large to render it difficult for him to dispense himself, to obtain the services of a dispenser whose fitness has been certified by competent authority, more especially as large numbers of young women are now available for the purpose at moderate terms. But the fatal case at Heaton Norris, assuming it to have occurred, was probably an accident, and no precautions will suffice wholly to exclude accidents from human affairs. A practitioner who ordinarily does his own dispensing, or who employs a qualified dispenser, may nevertheless in an emergency be compelled to obtain the help of some member of his household, who may make a mistake hardly to have been foreseen; and against such contingencies no precaution could avail. Accidents from inaccurate dispensing must at least be extremely rare, and it seems hardly likely that the Medical Council will see its way to any sweeping change for the sake of avoiding them. In all probability the public derives all necessary security from the liability of an employer for the acts or defaults of his servants.

